# Dietary Patterns Impact Temporal Dynamics of Fecal Microbiota Composition in Children With Autism Spectrum Disorder

**DOI:** 10.3389/fnut.2019.00193

**Published:** 2020-01-10

**Authors:** Kirsten Berding, Sharon M. Donovan

**Affiliations:** ^1^Division of Nutritional Sciences, University of Illinois, Urbana, IL, United States; ^2^Department of Food Science and Human Nutrition, University of Illinois, Urbana, IL, United States; ^3^Carl R. Woese Institute for Genomic Biology, University of Illinois, Urbana, IL, United States

**Keywords:** autism, microbiota stability, dietary patterns, nutrition, short chain fatty acids

## Abstract

Environmental factors such as diet are known influencers on gastrointestinal (GI) microbiota variability and some diseases are associated with microbial stability. Whether microbial variability is related to symptoms of Autism Spectrum Disorder (ASD) and how diet impacts microbial stability in ASD is unknown. Herein, temporal variability in stool microbiota in relation to dietary habits in 2–7 years-old children with ASD (ASD, *n* = 26) and unaffected controls (CONT, *n* = 32) was investigated. Fecal samples were collected at baseline, 6-weeks and 6-months. Bacterial composition was assessed using 16S rRNA sequencing. Short fatty acid (SCFA) concentrations were analyzed by gas chromatography. Nutrient intake was assessed using a 3-day food diary and dietary patterns (DP) were empirically derived from a food frequency questionnaire. Social deficit scores (SOCDEF) were assessed using the Pervasive Developmental Disorder Behavior Inventory-Screening Version (PDDBI-SV). GI symptoms were assessed using the GI severity index. Overall, temporal variability in microbial structure, and membership did not differ between the groups. In children with ASD, abundances of *Clostridiaceae*, Streptophyta, *and Clostridiaceae Clostridium*, varied significantly, and concentrations of all SCFAs decreased over time. Variability in community membership was negatively correlated with median SOCDEF scores. Additionally, Clostridiales, *Lactococcus, Turicibacter, Dorea*, and *Phascolarctobacterium* were components of a more stable microbiota community in children with ASD. DP1, characterized by vegetables, starchy vegetables, legumes, nuts and seeds, fruit, grains, juice and dairy, was associated with changes in species diversity, abundance of *Erysipelotricaceae, Clostridiaceae Clostridium*, and *Oscillospira* and concentrations of propionate, butyrate, isobutyrate and isovalerate in children with ASD. DP2 characterized by fried, protein and starchy foods, “Kid's meals,” condiments, and snacks was associated with variations in microbiota structure, abundance of *Clostridiaceae Clostridium*, and *Oscillospira* and changes in all SCFA concentrations. However, no association between microbial stability and SOCDEF or GI severity scores were observed. In conclusion, microbiota composition varies over time in children with ASD, might be related to social deficit scores and can be impacted by diet. Future studies investigating the physiological effect of the changes in specific microbial taxa and metabolites are needed to delineate the impact on ASD symptomology.

## Introduction

The gastrointestinal (GI) microbiota is increasingly being recognized for its ability to modulate host physiology, including host metabolism, immune function, as well as, behavior and cognition. A growing body of work has implicated that many neurological and psychiatric diseases can be associated with an altered microbial composition, including Autism Spectrum Disorder (ASD). Although it has previously been suggested that the GI microbiota under normal conditions is relatively stable, other studies have shown that even in adults the microbiota composition can be highly variable and changes over the course of a day (1) or weeks (2, 3) can occur. Thereby, microbial stability is defined as the “ability to respond to perturbations by resisting change and returning to the original state” (4). Higher microbial stability is associated with a healthier microbiota due to the ability to maintain bacterial function, to resist environmental stressors and protect against infection by pathogens (5–7). Some studies suggest that a stable individual microbial core containing predominant microbial taxa exists, whereas low abundance members may contribute more significantly to the variation in the microbiome (3, 8, 9). Likewise, the degree of variation in the microbiome might be specific to each individual, meaning that microbial taxa that are stable in one individual might undergo more variation in another individual (10). Understanding the long-term variability of the GI microbiota is important as resistance to environmental stressors and pathogen invasion, as well as, a rapid return to a baseline state after perturbation are key features of a healthy microbiome (5, 11). Indeed, previous studies have linked greater microbiota instability to certain disease states [e.g., Crohn's disease, irritable bowel syndrome (12–14)].

Changes in GI microbial composition over time can be attributed to environmental factors and diet has been identified as one of the most influential modifying factors. Although extreme shifts in the intake of dietary macronutrients and fiber are associated with short-term changes in the GI microbiota, most studies have found that these shifts are reversible and that the GI microbiota returns to a baseline composition (15, 16). On the other hand, habitual, longer-term dietary patterns might be more influential on microbial stability and a healthier long-term dietary pattern could promote a microbial profile that can protect against the development of certain diseases (17, 18). Previously, in healthy children a dietary pattern that was characterized by higher intakes of vegetables and lower intakes of snacks, sweets and dairy food was associated with greater microbial stability over a 6-month period, demonstrating a direct relationship between dietary patterns and temporal variability of the fecal microbiota (19).

Although increasing evidence is presented for a microbial dysbiosis in individuals with ASD, little is known about the temporal variability of the microbiota. Given the new evidence showing that specific microbes (e.g., *Clostridium* or *Desulfovibrio*) could contribute to some symptoms of ASD (20, 21), identifying long-term dietary patterns that could potentially promote the stability of a more beneficial microbiota composition could be important advances in the understanding of the microbiota-brain connection in ASD. Thus, the goal of this longitudinal observational study was to test the hypothesis that children with ASD will have more variability in their microbiota compared to unaffected controls over a 6 month period and that a dietary pattern characterized by higher intakes of healthy foods such as fruits, vegetables and grains will be associated with a more stable microbiota composition. We further hypothesized that a higher degree of microbial variability over time will be associated with more severe ASD symptoms.

## Materials and Methods

A detailed description of study design, study population, sample collection and analysis has previously been described (20) and is briefly described below. Children between 2- and 7-years-of-age diagnosed with ASD (ASD; *n* = 26) and age- and sex-matched controls (CONT, *n* = 32) were recruited between April 2016 and October 2017. Baseline samples were collected after recruitment and additional samples were collected 6-weeks and 6-months after baseline sample collection. Introduction of medication, pre- or probiotics as well as specialty diets (e.g., gluten-free/casein-free diet) was monitored throughout the study period. Participants provided oral assent and their legal guardians provided written consent in accordance with the ethical standards of the Institutional Review Board of the University of Illinois at Urbana-Champaign.

### Assessment of Microbiota Composition and Short Chain Fatty Acid Concentration

For each subject, freshly-voided morning stool samples were collected for the analysis of the fecal microbiota and short chain fatty acid (SCFA) concentrations as previously described (20).

#### Microbiome Sequencing

Microbial DNA was extracted from stool using a bead beating method followed by a combination of QIAamp Fast DNA Stool Mini Kit (Qiagen, Valencia, CA) and the FastPrep-24 System (MP Biomedicals, Carlsbad, CA) as previously described (22). The V3 to V4 regions (ca. 430 bp) of 16S rRNA gene was amplified using established primers (forward, 5′-GTGCCAGCMGCCGCGGTAA−3′ and reverse, 5′-GGACTACHVGGGTWTCTAAT−3′) using the Fluidigm Access Array System and sequenced using Illumina MiSeq bulk V2 chemistry at the W. M. Keck Center at the University of Illinois, Urbana-Champaign, a previously described (23). The 16S rRNA sequences were processed and analyzed using the QIIME 1.9.1 bioinformatics package (24, 25). Sequences were demultiplexed and clustered into operational taxonomic units (OTUs) using closed-reference OTU picking with default parameters against the Greengenes 13_8 reference OTU database at a 97% similarity level. Singletons and OTUs with an abundance lower than 0.005% were removed prior to rarefying to a sampling depth of 49,446 sequences per sample for subsequent analysis. α- and β- diversity were calculated using QIIME. Taxonomy summary was performed using the core diversity script in QIIME.

#### qPCR

Bacterial genomic DNA was analyzed for total bacteria, *Lactobacillus* spp., *Bifidobacterium* spp. *Prevotella, Clostridium perfringens, C. difficile* as well as methylmalonyl-CoA decarboxylase (mmdA) gene in the succinate pathway of propionate production and the butyryl-CoA:acetate CoA acyltransferase (BCoAT) gene in the butyrate production pathway.

#### Short Chain Fatty Acids

Sample preparation for and analysis of SCFA concentrations were performed as previously described (22).

### ASD Symptoms Severity Assessment

Parents were asked to complete the Pervasive Development Disorder Behavior Inventory Screening Version (PDDBI-SV) (PAR, Inc., Lutz, FL) in order to assess the social deficit symptom severity. The answers are scored by summing the ratings and yield an overall T score for the severity of ASD symptoms. A T-score of 50 is defined as typical of children with ASD. As the SOCDEF score increases, social communication skills worsen and challenging behaviors increase (26).

### Assessment of Gastrointestinal Symptoms and Stool Consistency

The severity of GI symptoms was assessed for constipation, diarrhea, stool smell, flatulence, and abdominal pain in an adapted version of the GI Severity Index. This version has previously been used in a study investigating GI health in children with ASD (27). Additionally, average stool consistency was assessed using the Bristol Stool Chart (28).

### Assessment of Dietary Intake

Information on dietary intake was assessed using a 3-day food diary and the Youth and Adolescent Food Frequency Questionnaire (YAQ). To monitor short-term nutrient intake prior to fecal sample collection, a 3-day dietary food record was completed the 3 days prior to each sample collection. The food diary data were analyzed using the Nutrition Data System for Research (NDSR, Minneapolis, MN, 2014) software to assess nutrient intake and for comparison to recommended intakes (i.e., Recommended Daily Allowance, Adequate Intake). The YAQ containing 156 food items was completed by the parents to estimate their child's usual food and beverage intake over the past year (29). To estimate the number of servings of any food group, each response was converted to the corresponding frequency factor (Harvard T.H. Chan School of Public Health Nutrition Department's Food Group Serving Table) and summed over all the food items to get the average servings of a specific food group per day.

In addition, information on food intolerances, food avoidance and nutritional supplement use, including pre- and probiotics, and medical prescriptions was collected in an online questionnaire.

### Statistics

All data were analyzed using SAS 9.4 (SAS Institute, Cary, NC). The 16S rRNA sequences were processed and analyzed using QIIME 1.9.1 bioinformatics software and baseline dietary patterns (DP) derived from the YAQ using Principal Component and Factor Analysis with Varimax rotation as previously described (19). Scores for DP were calculated for each participant and stratified as either falling above or below the median. Data in the ASD group was analyzed for significant differences between the two groups based on DP (above vs. below median). Variability in diversity measures (α- and β-diversity) was assessed for participants who provided all three sample (ASD *n* = 22; CONT *n* = 29). Variability of α-diversity (within an individual over time) was assessed by calculating the coefficient of variation (CV = standard deviation/mean) for Chao 1 index, observed OTUs and Shannon index (30). Higher values indicated a more variable community. The median weighted and unweighted UniFrac distances for each individual over time were calculated to determine the variability in community composition over time (3). Higher median values correspond to more variability whereas lower median values are indicative of a more stable microbial community. For both groups, the study population was divided into stability classes (falling above or below the median based on median weighted UniFrac distances) in order to determine which bacteria or dietary measures could contribute to the stability or instability of the overall composition (3, 7). Children falling above the median were denoted as less stable whereas children below the median were denoted as more stable. Differences in outcome measures were analyzed using mixed models including participant as a repeated effect. Model fit was assessed using the Chi-square-to-df ratio. Values <2 were indicative of an appropriate model fit (1). Factors known to influence the microbiota composition including age, gender, body mass index (BMI), season of sample collection, change in use of medications, probiotics or specialty diet, as well as, food groups and nutrient intake that changed over the 6-month period were included as covariates. Spearman correlation was used to investigate relationships between variability and ASD symptoms. Data are expressed as median (IQR) or mean ± SD. Level of significance was set at *p* ≤ 0.05 and *p* ≤ 0.10 was considered a trend. All p-values reported are FDR corrected for multiple comparisons using the Bonferroni-correction.

## Results

### Enrollment and Attrition Rate of Study Population

From the 32 children enrolled in the CONT group, all subjects provided samples at the 6-week time point and 29 subjects provided samples at the 6-month time point. In the ASD group, from the 26 children enrolled at baseline, 25 children provided samples at the 6-week time point and 22 at the 6-month time point. Participant characteristics at baseline are previously described (20).

### Introduction of Medication, Specialty Diets, or Probiotics

Three children with ASD began medication during the study, including Clonidine, Conerta (stimulant), and Namenda (cognitive enhancing medicine). Three CONT children took medication over the study period, including cold relief medication or antibiotics. Regarding specialty diets, no children in the CONT group started a specialty diets. In the ASD group, four children were following a specialty diet at the 6-week sample collection and seven children at the 6-month time point. These diets included the gluten-free/casein-free diet and other exclusions diets (i.e., decrease sugar alcohols and fructose). Probiotics were not introduced in either of the groups.

### GI Symptoms, Stool Consistency, and ASD Symptoms

GI symptoms, stool consistency as measured by the Bristol stool chart and SOCDEF T-scores did not change over the 6-month study period.

### Changes in Nutrient and Food Group Intake From Baseline to 6-Month Post-baseline

Nutrient intake from the 3-day food records were analyzed for changes over the 6-month period to determine the potential impact of diet on the temporal variability of the GI microbiota. In the ASD group, a decrease in intake of vitamin A (*p* = 0.02) and vitamin E (*p* = 0.08), was observed. In the CONT group, no significant changes in nutrients were observed. Regarding intake of food groups, no changes were observed in the ASD group. In the CONT group, intake of starchy vegetables (*p* = 0.04) and protein foods (*p* = 0.03) increased from baseline to 6-week and 6-month post-baseline with the greatest intake at the 6-week time point (Supplemental Table 1).

### Temporal Microbial Diversity and Changes in Microbial Taxa

α- and β-diversity, bacterial abundance at the phyla level, expression of mmDA and BCoAT as well as the count of bacteria assessed by qPCR did not differ between the time points in either group (data not shown).

**Figure 1:**
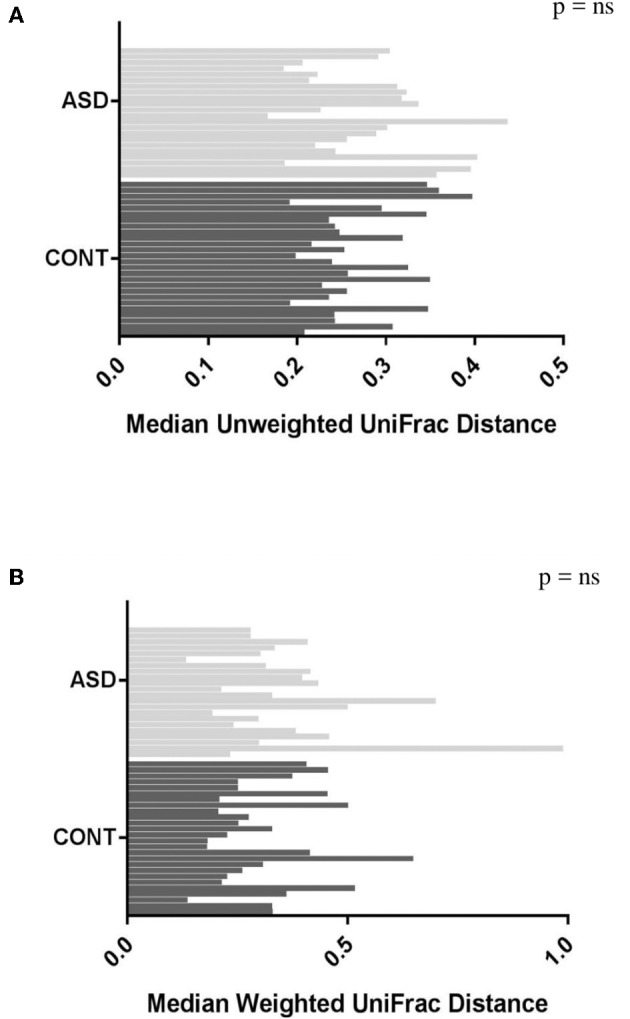
Plots of variability measured by unweighted **(A)** and weighted **(B)** UniFrac distance in children with ASD and CONT. Temporal variability in β-diversity did not differ between the groups.

**Figure 2:**
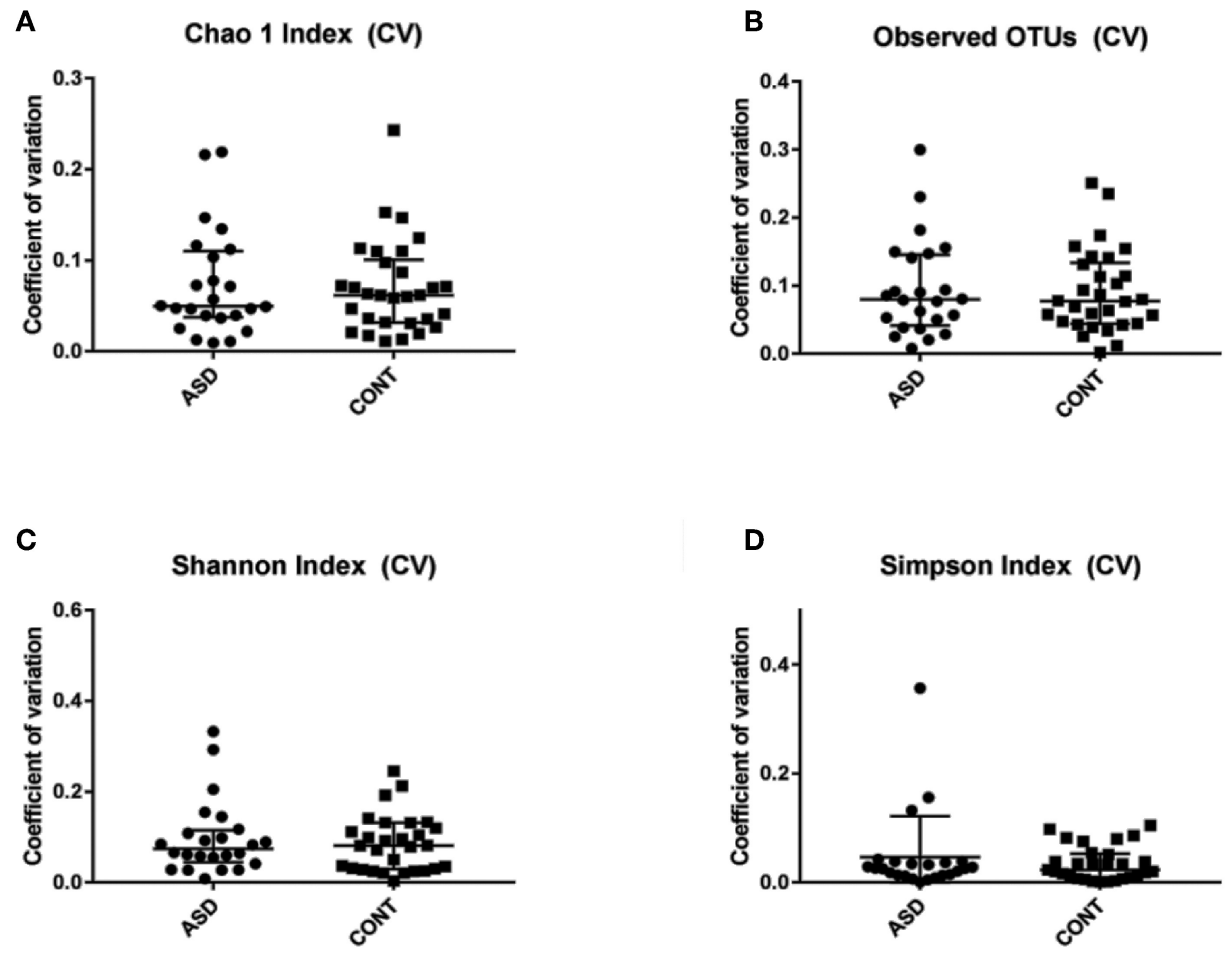
Temporal variability measured by coefficient of variation (CV) in α-diversity measures. Data expressed as median (IQR). There was not difference between the groups. CV is shown for **(A)** Chao 1 Index, **(B)** Observed OTUs, **(C)** Shannon Index and **(D)** Simpons Index. CV - Coefficient of variation; ASD - children with Autism spectrum disorder; CONT - control group; Data expressed as mean ± SEM.

When comparing temporal variability between children with ASD and unaffected controls, no differences in variability in community membership (unweighted UniFrac) and structure (weighted UniFrac) (Figure 1) as well as temporal variability within each group quantified by the coefficient of variation (CV) of species richness (Chao 1 Index and observed OTUs) and diversity (Shannon and Simpson Index) (Figure 2) did not differ. Analyzing changes in specific microbial taxa showed that different bacteria changed over a 6-month period in each group. In ASD children, abundance of Streptophyta (*p* = 0.07) increased whereas abundances of *Clostridiaceae* (*p* = 0.01), and *Clostridium* (*p* = 0.003) decreased over the 6-month study period, with the highest abundance of *Clostridiaceae* being observed at the 6-week time point. In CONT children, the abundance of *Enterobacteriaceae* was increased (*p* = 0.01) and *Enterococcus* tended (*p* = 0.08) to increase over the 6-month study period (Table 1).

**Table 1:** Measures of relative abundance of bacterial genera in feces that differed between time points in children with ASD and CONT.

**Bacterial taxa (% of sequences)**	**Baseline (*n =* 26)**	**6-weeks p.-b. (*n =* 25)**	**6 months p.-b. (*n =* 22)**
**ASD**			
**Bacterial order**			
Streptophyta	0 (0–0.001)	0 (0–0.005)	0.002 (0–0.02)^†^
**Bacterial family**			
Clostridiaceae	0.28 (0.14–1.5)	0.53 (0.16–1.47)	0.25 (0.08–0.49)^†^
**Bacterial genus**			
*Clostridaceae_Clostridium*	0.33 (0.05–0.66)	0.19 (0.07–0.42)	0.06 (0.03–0.14)^*^
**CONT**			
**Bacterial taxa (% of sequences)**	**Baseline (*****n=*** **32)**	**6-weeks p.-b. (*****n=*** **32)**	**6 months p.-b. (*****n=*** **29)**
**Bacterial family**			
Enterobacteriaceae	0.02 (0.007–0.12)	0.02 (0.01–0.39)	0.03 (0.005–0.11)^†^
**Bacterial genus**			
*Enterococcus*	0 (0–0.001)	0 (0–0.002)	0.001 (0–0.002)^†^

Data expressed as median (IQR); within same group and row, significant at FDR-corrected

* p ≤ 0.05;

† *p ≤ 0.1; p.-b., post-baseline*.

### Association Between Microbial Variability and ASD Symptoms

**Figure 3:**
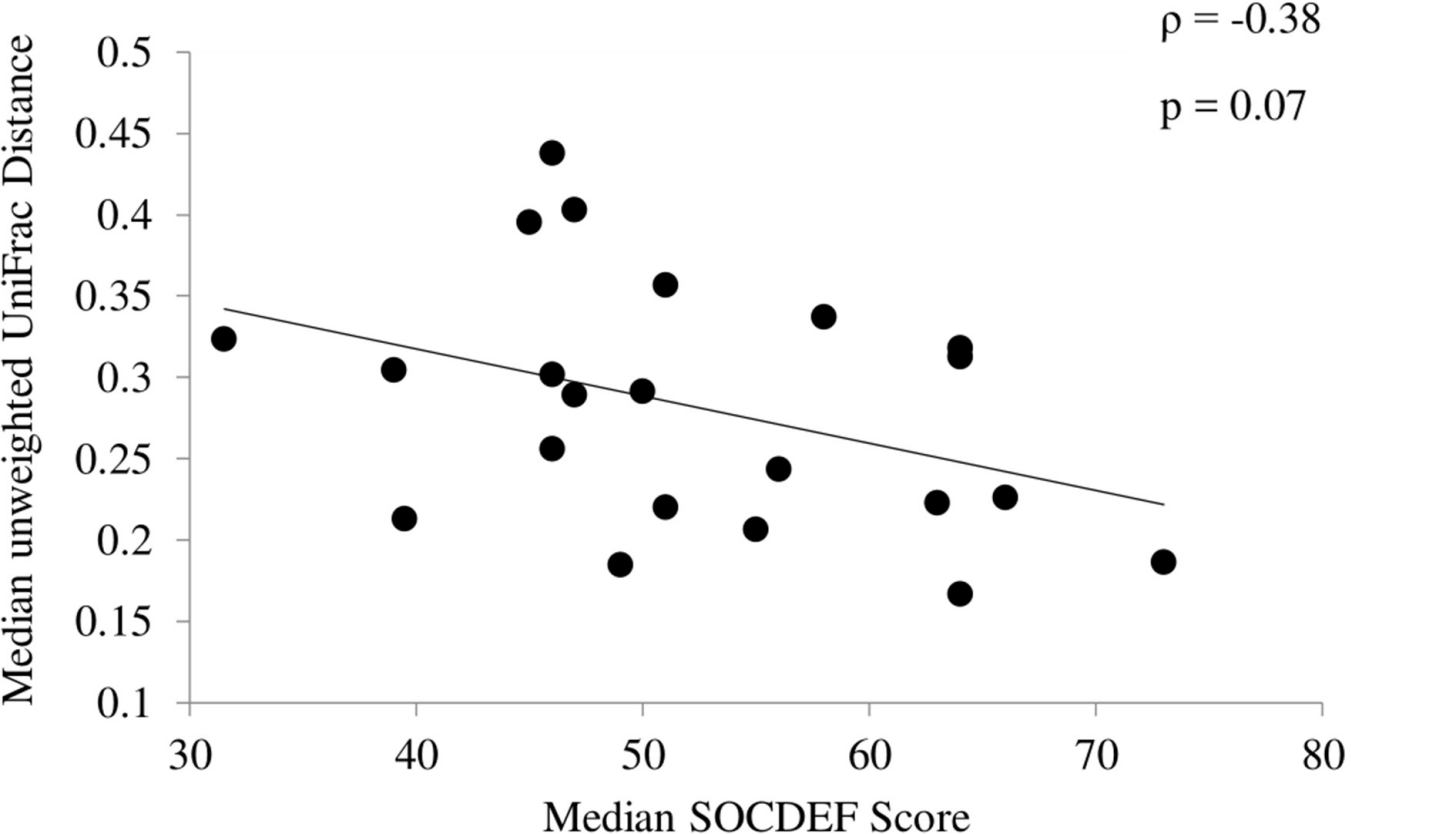
Association between social deficit scores and unweighted UniFrac distance. Children with ASD and less variability in community membership measured by the median unweighted UniFrac distance had higher social deficit scores.

In children with ASD, variability based on community membership (unweighted UniFrac) tended to be negatively correlated (ρ = −0.38; *p* = 0.07) with median SOCDEF scores over the 6-month period (Figure 3). The diversity within each child with ASD (based on CV for α-diversity measures) was not associated with SOCDEF scores.

### SCFA Concentrations From Baseline to 6-Month Post-baseline

**Figure 4:**
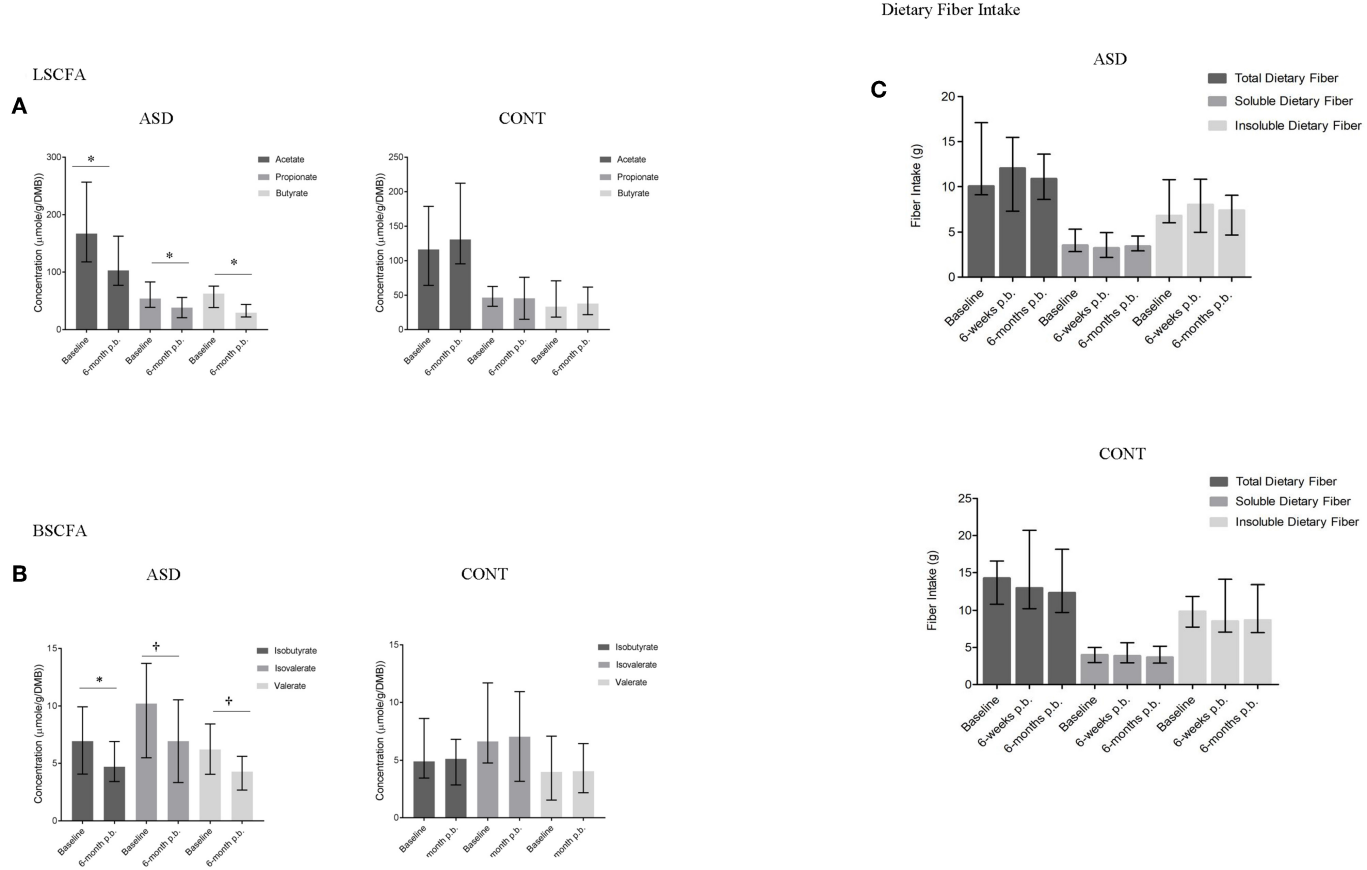
SCFA concentrations and dietary fiber intake at baseline and 6-months post-baseline (p.-b.) in children with ASD (ASD) and unaffected controls (CONT). Concentration of all SCFAs decreased in children with ASD whereas SCFA concentrations in CONT remained stable. **(A)** LSCFA and **(B)** BSCFA concentrations at baseline and 6-months post-baseline for ASD and CONT; **(C)** Dietary fiber intake at baseline and 6-months post-baseline for ASD and CONT; LSCFA, linear short chain fatty acids (acetate, propionate, butyrate); BSCFA, branched short chain fatty acids (Isobutyrate, isovalerate, valerate); p.b. - post-baseline; data expressed as median (IQR); ^*^p ≤ 0.05; ^†^*p* ≤ 0.1.

In ASD, a statistically significant decrease in the concentration of acetate (*p* = 0.01), propionate (*p* = 0.05), butyrate (*p* = 0.02), and isobutyrate (*p* = 0.01) and a trend for a decrease in isovalerate (*p* = 0.05) and valerate (*p* = 0.09) concentrations was observed over time. In CONT, SCFA concentrations did not change over the 6-month study period (Figures 4A,B). Dietary fiber consumption did not change in either group over the 6-months study period (Figure 4C).

### Bacterial Taxa and Dietary Factors Contributing to Microbial Variability

**Figure 5:**
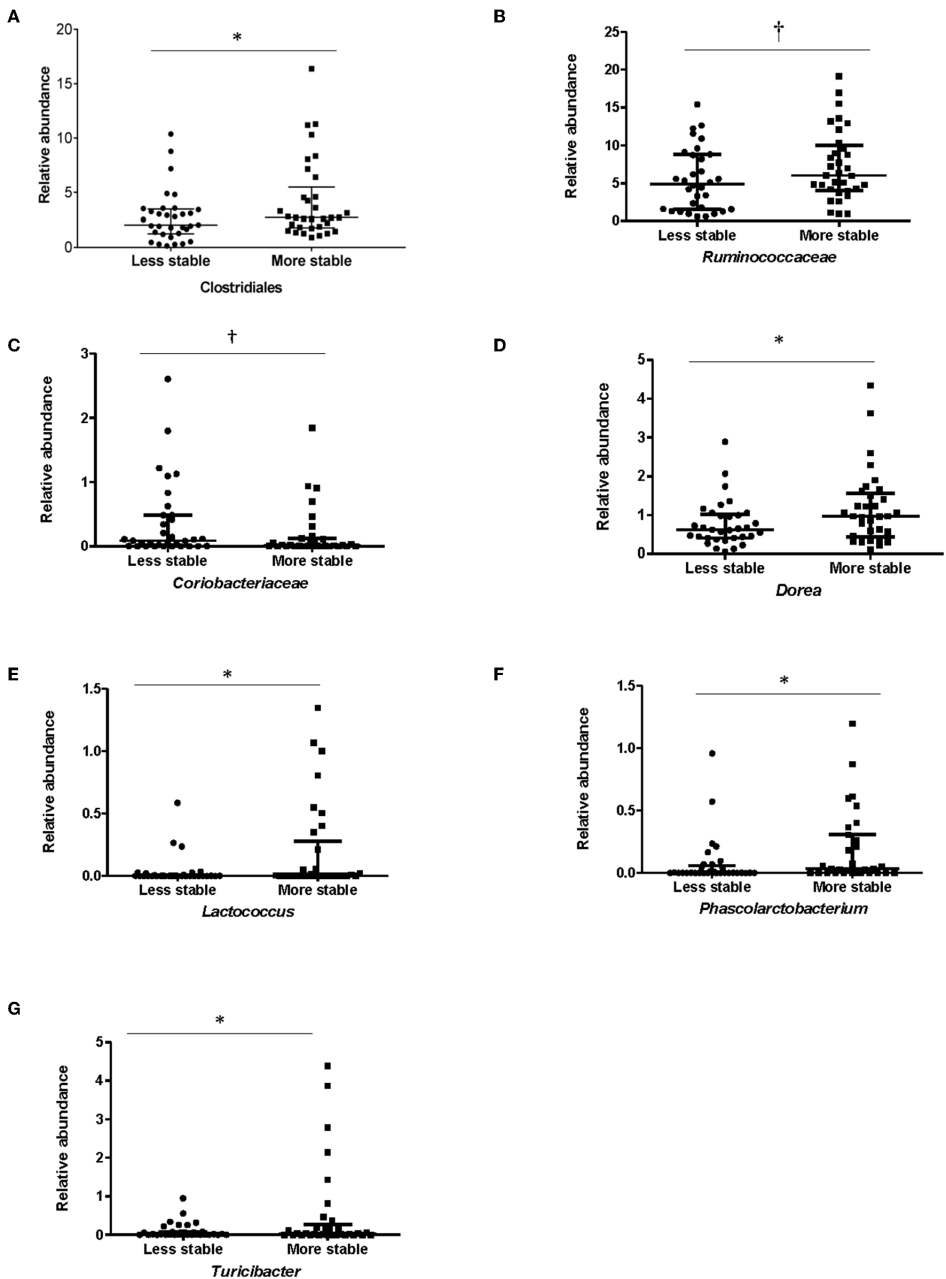
Bacterial taxa that differed significantly among stability categories in children with ASD. Relative abundance for **(A)** *Clostridiales*, **(B)** *Ruminococcaceae*, **(C)** *Coriobacteriaceae*, **(D)** *Dorea*, **(E)** *Lactococcus*, **(F)** *Phascolarctobacterium*, and **(G)** *Turicibacter* differed between more and less stable categories in children with ASD. Data expressed as median (IQR); ^*^p ≤ 0.05; ^†^*p* ≤ 0.1; Individuals in the ASD group were assigned to a category of stability based on falling above or below the median of median weighted UniFrac distances.

In order to determine whether specific bacterial taxa could contribute to a more or less stable microbial structure in both groups, differences in the average relative abundance of bacterial taxa and dietary factors between children dichotomized by stability category (more stable vs. less stable) were assessed. In children with ASD, Clostridiales (*p* = 0.05), (*p* = 0.05), *Lactococcus* (*p* = 0.002), *Turicibacter* (*p* = 0.04), *Dorea* (*p* = 0.04), and *Phascolarctobacterium* (*p* = 0.05) were more abundant in children with ASD whose microbiota structure was temporally more stable over a 6-month period (Figure 5). Measures of bacterial richness and diversity as well as SCODEF T-score and GI symptoms severity scores did not differ based on stability category.

Regarding dietary intake, children with ASD that had a more stable microbial community had higher intake of total protein (*p* = 0.06) whereas children with ASD categorized as having lower microbial stability had higher intake of condiments (*p* = 0.03) (Table 2).

**Table 2:** Nutrient and food group contributing to stability categories in children with ASD.

	**Less stable (*n =* 13)**	**More stable (*n =* 13)**
**Food group (servings/day)**^**a**^		
Fish	0 (0–0.14)	0.04 (0–0.14)^†^
Condiments	0.36 (0–0.5)	0.08 (0–0.4)^*^
**Nutrient**^**b**^		
Total protein (g)	43 (34–49)	52 (31–68)^†^

Data expressed as Median (IQR); FDR-corrected

* p ≤ 0.05;

† *p ≤ 0.1; Individuals in the ASD group were assigned to a category of stability based on falling above or below the median of median weighted UniFrac distances*.

a *Food groups derived from food frequency questionnaire*.

b *Nutrient intake derived from 3-day food diary*.

CONT children with a more stable microbial community tended to have higher species diversity as measured by the Shannon index (*p* = 0.1) compared to children with a less stable microbiota. Verrucomicrobia (*p* = 0.05), *Enterobacteriaceae* (*p* = 0.09), and *Akkermansia* (*p* = 0.02) were more abundant in CONT children with a more unstable microbiota. On the other hand, *Adlercreutzia* (*p* = 0.04), *Faecalibacterium* (*p* = 0.07), and *Sutterella* (*p* = 0.01) and *Bilophila* tended (*p* = 0.06) to be more abundant in CONT children with a more stable microbiota community over the 6-month study period. Regarding dietary intake, CONT children with a more stable microbiota had higher intakes of whole grains (*p* = 0.04), vegetables (*p* = 0.05), fish (*p* = 0.01), and condiments (*p* = 0.03) and a tendency (*p* = 0.09) for higher intake of sweetened beverages, whereas children with a less stable microbiota had higher intakes of Kid's meals (*p* = 0.03) (Supplemental Table 2).

### Nutrient and Food Group Intake, Microbiota Stability, and SCFA Concentrations Over 6-Month Period Based on Baseline DP in Children With ASD

In order to determine whether microbial stability was impacted by baseline dietary patterns, changes in microbiota abundance and SCFA concentrations in children with ASD were investigated based on dietary patterns. The process for determining dietary patterns was as previously described (20).

#### Dietary Pattern 1

In children above the median in DP1, intake of servings per day of fruit (*p* = 0.05) and added sugars (*p* = 0.05) decreased and servings of vegetables (*p* = 0.06), and total carbohydrates (*p* = 0.09) tended to decrease over the 6-month study period (Table 3). Regarding the microbial variability composition, no difference in temporal variability of α-diversity community membership (unweighted UniFrac), structure (weighted UniFrac), or SCFA concentrations was observed (Table 4; Supplemental Figure 1; Supplemental Table 3), but relative abundance of *Erysipelotrichaceae* (*p* = 0.05) increased and abundance of *Clostridiaceae Clostridium* (*p* = 0.03) decreased over the study period (Table 4).

**Table 3:** Changes in nutrient intake baseline to 6 months in children with ASD based on DP.

	**Above Median**			**Below Median**		
	**Baseline**	**6-weeks p.-b**.	**6 months p.-b**.	**Baseline**	**6-weeks p.-b**.	**6 months p.-b**.
**(A) Dietary pattern 1**						
**Food Group (servings/day)**^**a**^						
Fruit	3.0 (2.7–3.6)	2.5 (1.6–2.7)	1.9 (1.4–2.7)^*^	1.0 (0.5–2.3)	1.1 (0.6–2.1)	1.8 (0.8–2.6)
Vegetables	1.7 (0.9–2.4)	0.7 (0.6–1.1)	0.9 (0.7–1.3)^†^	0.3 (0.08–0.7)	0.6 (0.4–1.3)	0.5 (0.2–0.9)
Legumes, nuts and seeds	0.4 (0–0.8)	0.08 (0–0.14)	0.1 (0–0.3)	0.1 (0.1–0.16)	0.1 (0.1–0.3)	0.22 (0.1–0.9)^*^
Refined carbohydrates	1.1 (0.9–1.7)	1.02 (0.9–1.2)	1.3 (0.8–1.4)	0.9 (0.5–1.1)	1.4 (1.2–1.5)	1.1 (0.9–1.9)^*^
**Nutrient**^**b**^						
Total carbohydrate (g)	194 (143–220)	173 (162–194)	133 (102–182)^†^	173 (151–209)	141 (112–254)	182 (117–209)
Added sugars (g)	31 (24–42)	36 (19–42)	21 (12–26)^†^	36 (19–42)	35 (22–55)	20 (17–55)
**(B) Dietary pattern 2**						
Refined Carbohydrates	0.9 (0.8–1.4)	1.2 (1.0–1.3)	0.9 (0.7–1.2)	0.9 (0.6–1.2)	1.1 (0.9–1.4)	1.5 (0.9–1.8)^†^
**Nutrient**^**b**^						
Vitamin A (μg)	412 (282–565)	395 (198–488)	252 (96–445)^†^	441 (278–651)	315 (234–475)	275 (218–321)
Sodium (mg)	1985 (1687–2228)	1891 (1635–2245)	1445 (1199–2133)^†^	1891 (1635–−2245)	1660 (1461–2180)	1825 (1470–2019)
Vitamin E (μg)	5.8 (4.2–7.9)	5.7 (3.9–6.8)	5.4 (3.5–6.5)	6.5 (4.5–9.5)	5.6 (4.2–7.4)	4.2 (3.5–5.7)^†^

Data expressed as median (IQR); FDR-corrected

* p ≤ 0.05;

† *p ≤ 0.1; p.b., post-baseline*.

a *Food groups derived from food frequency questionnaire*.

b *Nutrient intake derived from 3-day food diary*.

**Table 4:** Changes in relative abundance of bacterial genera and SCFA based on DP in children with ASD.

	**Above Median**			**Below Median**		
	**Baseline**	**6-weeks p.-b**.	**6 months p.-b**.	**Baseline**	**6-weeks p.-b**.	**6 months p.-b**.
**(A) Dietary pattern 1**						
**Bacterial richness/diversity**						
Shannon Index	5.3 (4.7–5.5)	5.2 (4.6–5.6)	5.1 (4.7–5.6)	5.7 (5.2–6.1)	5.3 (5.0–5.8)	4.8 (4.3–5.3)^†^
**Bacterial taxa (% of sequences)**						
Erysipelotrichaceae	0.3 (0.12–0.98)	0.98 (0.22–1.23)	0.9 (0.33–1.44)^†^	0.51 (0.22–1.29)	0.59 (0.51–0.92)	0.25 (0.13–1.35)
*Clostridium*	0.33 (0.11–0.66)	0.11 (0.07–0.33)	0.09 (0.03–0.11)^†^	0.23 (0.05–0.49)	0.26 (0.09–0.50)	0.05 (0.03–0.11)^†^
*Oscillospira*	0.43 (0.25–0.52)	0.35 (0.23–0.44)	0.32 (0.27–0.65)	0.37 (0.29–0.56)	0.15 (0.11–0.37)	0.14 (0.07–0.25)^*^
**SCFA**						
Acetate	155 (214–229)	·	98 (76–136)	173 (131–242)	·	126 (84–211)
Propionate	50 (39–77)	·	30 (23–41)	55 (51–90)	·	38 (39–59)^†^
Butyrate	49 (37–74)	·	29 (22–38)	65 (42–76)	·	29 (23–47)^*^
Valerate	6.8 (4.7–8.1)		4.9 (1.4–5.5)	6.8 (4.7–8.1)	·	3.9 (2.9–5.6)
Isobutyrate	6.2 (4.2–7.6)	·	5.5 (4.7–7.1)	7.8 (4.5–10)	·	3.9 (2.3–4.6)^†^
Isovalerate	8.5 (5.5–11)	·	7.6 (6.7–11)	11.7 (6.3–15)	·	4.8 (3.2–6.4)^†^
**(B) Dietary pattern 2**						
**Bacterial taxa (% of sequences)**						
Erysipelotrichaceae						
*Clostridium*	0.23 (0.05–0.49)	0.28 (0.06–0.44)	0.05 (0.03–0.14)^†^	0.42 (0.17–0.66)	0.13 (0.08–0.39)	0.07 (0.02–0.16)^†^
*Oscillospira*	0.38 (0.37–0.52)	0.28 (0.13–0.40)	0.17 (0.13–0.29)^†^	0.43 (0.21–0.56)	0.34 0.17–0.40)	0.34 (0.26–0.67)
**SCFA**						
Acetate	200 (131–255)	·	150 (78–211)	143 (110–246)	·	102 (74–127)^*^
Propionate	68 (51–90)	·	38 (28–59)^†^	50 (39–61)	·	29 (23–42)^*^
Butyrate	67 (41–76)	·	26 (23–43)^*^	49 (37–74)	·	29 (10–49)
Valerate	7.8 (6.5–10.8)		4.5 (2.9–5.6)^*^	4.1 (0.9–5.6)		4.1 (0.9–5.6)
Isobutyrate	9.4 (6.2–11.5)	·	4.6 (2.3–6.9)^*^	5.4 (3.7–7.2)	·	5.1 (3.8–6.9)
Isovalerate	13 (8–15.5)	·	6.4 (3.2–8.4)^*^	6.7 (5.5–10.6)	·	7.5 (4.7–10.4)

Data expressed as median (IQR); within same group and row, significant at FDR-corrected

* p ≤ 0.05;

† *p ≤ 0.1; p.b., post-baseline*.

More changes in nutrient and food group intake and microbiota composition were observed in children with ASD below the median in DP1. An increase in servings per day of legumes, nuts and seeds (*p* = 0.1), and refined carbohydrates (*p* = 0.01) was observed in children below the median in DP1 (Table 3). Regarding the microbiota composition, species diversity based on Shannon index (*p* = 0.002), decreased in children below the median in DP1. Additionally, abundance of *Clostridiaceae Clostridium* (*p* = 0.02), and *Oscillospira* (*p* = 0.04) decreased over the 6-month study period in children below the median in DP1 (Table 4). Lastly, concentrations of butyrate (*p* = 0.02) decreased and propionate (*p* = 0.08), isobutyrate (*p* = 0.09) and isovalerate (*p* = 0.06) tended to decrease in children below the median in DP1 (Table 4).

#### Dietary Pattern 2

**Figure 6:**
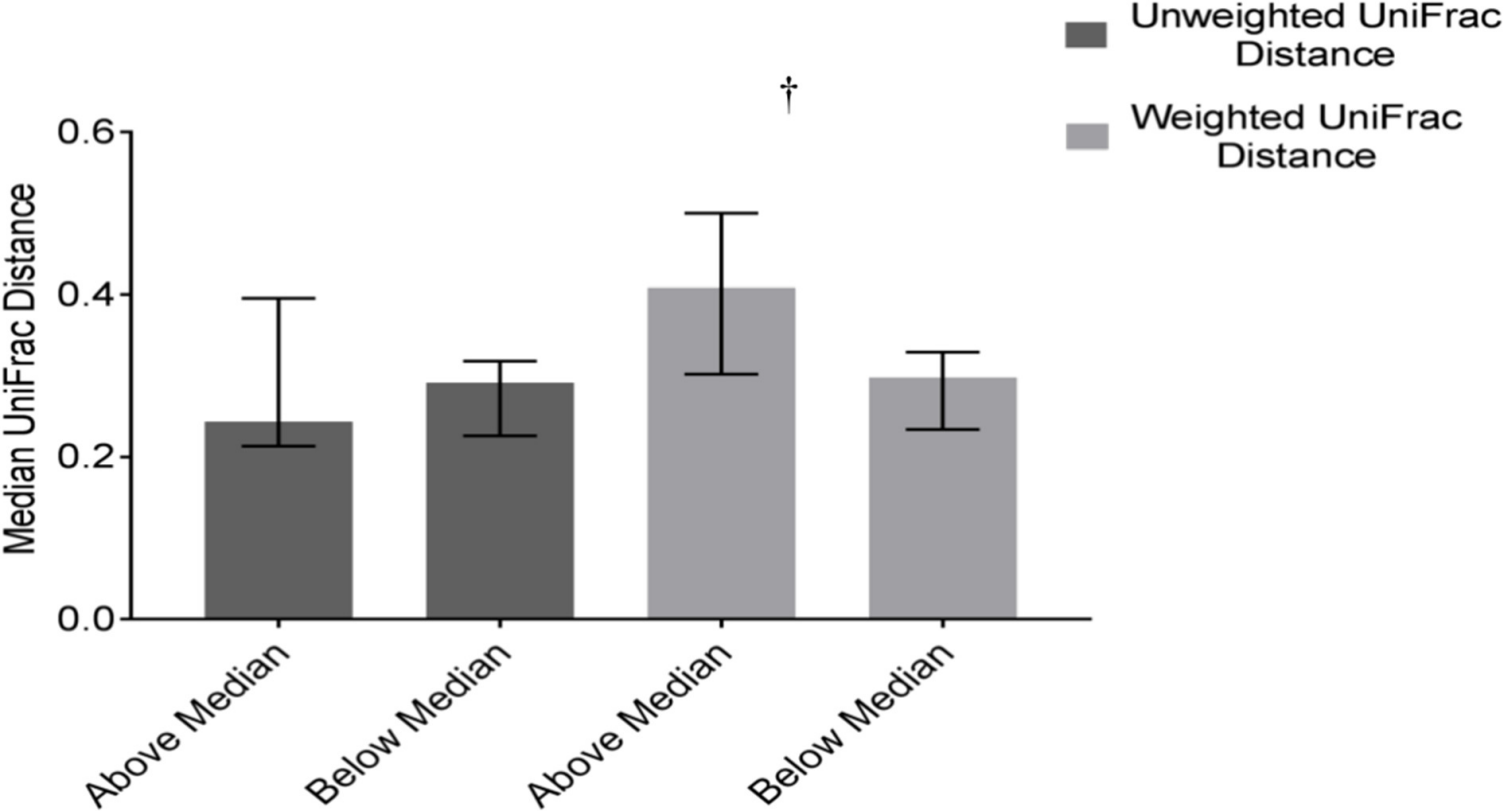
Differences in weighted and unweighted UniFrac distances based on DP2 in ASD. Children with ASD falling above the median in DP2 tended to have higher variability in community structure measured by weighted UniFrac distance than children below the median; Data expressed as median (IQR); ^†^*p* ≤ 0.1.

Community structure variability (median weighted UniFrac) tended (*p* = 0.07) to differ based on DP2. Children above the median in DP2 tended to have higher variability (higher median values) compared to children below the median (Figure 6). There was no difference based on DP2 in median unweighted UniFrac or temporal variability of α-diversity (Figure 6, Supplemental Table 3).

Children with ASD falling above the median in DP2 tended to have decreased intakes of vitamin A (*p* = 0.1) and sodium (*p* = 0.1) (Table 3). Regarding the microbiota composition a decrease in the abundance of *Clostridiaceae Clostridium* (*p* = 0.01) and *Oscillospira* (*p* = 0.04) was observed. Additionally, concentrations of butyrate (*p* = 0.02), isobutyrate (*p* = 0.02), isovalerate (*p* = 0.02) and valerate (*p* = 0.04) decreased and propionate tended (*p* = 0.08) to decrease in children above the median in DP2 (Table 4).

Children with ASD falling below the median in DP2 tended to have increased (*p* = 0.09) intakes of refined carbohydrates and decreased (*p* = 0.1) intakes of vitamin E (Table 3). The microbial profile was characterized by a trend for decreases in *Clostridiaceae Clostridium* (*p* = 0.06). Acetate (*p* = 0.04) and propionate (*p* = 0.02) concentrations also decreased significantly in children falling below the median in DP2-ASD (Table 4).

SOCDEF and total GI severity scores did not significantly change over time in either dietary pattern in children with ASD.

## Discussion

Increasing evidence supports microbial dysbiosis in children with ASD and some studies suggest that specific bacterial taxa might be associated with some symptoms of ASD (20, 21, 31). Additional evidence indicates that microbial instability could be linked to some diseases such as Crohn's or Irritable Bowel Disease (8, 12). However, to the best of our knowledge no studies have investigated microbial stability in children with ASD and whether it could be linked to symptom severity. Additionally, various environmental factors, such as host health (32), genetics (33), age, and diet (19) are known to influence the abundance of bacterial taxa. Achieving a balanced dietary intake can often be challenging in children with ASD (34) and dietary interventions (i.e., gluten-free/casein-free diet) are commonly used by parents in an effort to alleviate some symptoms of ASD (35). However, how habitual dietary patterns are potentiality correlated with the microbiota stability in children with ASD is unknown.

To address this gap in the literature, three fecal samples as well as information on diet were collected from children with ASD and unaffected controls over a 6-month period. Additionally, symptoms of children with ASD were assessed at each time point in order to investigate microbial stability in relation to dietary patterns and symptoms of ASD. Changes in the microbiota composition with different bacterial taxa contributing to a stable microbiota were observed in both groups. Although microbial stability was associated with baseline dietary patterns and food intake, the severity of social deficits and GI symptoms were not associated with a microbial profile linked to baseline dietary patterns. Thus, this study provides preliminary evidence that that long-term eating patterns in children with ASD impact the temporal variability of the microbiota; additional research is needed to further delineate the potential impact on symptoms of ASD.

Recent studies have linked microbial stability to certain diseases and mood (8, 12–14, 36, 37). Contrary to our hypothesis, neither the overall diversity within an individual, nor the variability in community structure and membership across time differed between children with ASD and unaffected controls. Likewise, the previously reported temporal changes in the α- and β-diversity of study populations (3) were not observed in either group. Yet, other studies have reported that the microbial community might be relatively stable over time (38–40). Even though overall microbial structure did not change significantly, changes in individual bacteria as well as in microbial metabolites were observed in this cohort.

In children with ASD, a trend toward changes in the abundance of bacteria with potential interest to ASD symptomology (e.g., *Clostridiaceae, Clostridiaceae Clostridium*) were detected. Interestingly, the variation in the abundance of *Clostridiaceae Clostridium* seemed to be independent of dietary factors. Higher abundances of *Clostridium* are often observed in the ASD population (41, 42) and it has been hypothesized that *Clostridium* contributes to ASD symptoms due to the production of entero- and neurotoxins (43). Additionally, the abundance of Clostridiales was increased in children with ASD who developed GI symptoms at or around the same time of ASD diagnosis, suggesting that this bacterial order could potentially contribute to symptom development (44). Previous studies have reported that *Clostridiaceae*, a family within the order of Clostridiales, was more prevalent in adults with a more variable microbiota composition over a 3-month period (3). Surprisingly, in this cohort, Clostridiales was more abundant in children with ASD and a more stable microbiota. Since Clostridiales has been associated with ASD symptomology it could be hypothesized that a stable microbiota that is high in bacteria within the Clostridiales order in children with ASD might be less beneficial and associated with persistent ASD symptomology. However, it is important to note that genera and species within Clostridiales also include commensals with potentially beneficial effects (45–49). Thus, future studies at the species level are required to delineate whether a stable microbiota is more beneficial in children with ASD. Likewise, further research is warranted to elucidate which species within the Clostridiales order could potentially promote a more stable microbiota in children with ASD. Other microbial genera with a potentially stabilizing effect in the ASD population (i.e., *Lactococcus, Turicibacter, Phascolartobacterium*) have been described as beneficial symbionts. Lactic acid bacteria, such as *Lactococcus*, could have beneficial functions through production of anti-microbial compounds, modulation of the host's immune response (50, 51) and counteracting the virulence factor of pathogenic bacteria such as *Staphylococcus aureus* (52). Low abundance of *Phascolartobacterium* was associated with the presence of inflammation in IBD (53) suggesting potential anti-inflammatory effects of these genera.

In the unaffected control group, different bacterial taxa were identified as stabilizing and destabilizing bacteria and similar to previously published literature, species diversity was associated with a more stable microbiota over the 6-month period (3). Surprisingly, this relationship was not observed in children with ASD. Thus, further investigation of this association is warranted, but it could indicate that diversity might affect stability differently depending on the health state of the host. Contrary to our hypothesis we also observed that children with ASD who have less changes in presence and absence of OTUs (based on unweighted UniFrac distance) had higher social deficit scores. Although less variability is considered to be more beneficial due to the ability to maintain bacterial functions over time and to resist pathogen invasion (6), less variability in ASD could correspond to a constant presence of pathogenic bacteria that could affect symptomology. The physiological significance of these bacterial changes observed in this ASD population, especially *Clostridiaceae Clostridium*, is unknown. Here, changes in the symptom severity of social deficits or GI symptoms did not change over the 6-month period. Thus, future studies potentially collecting samples more frequently, including a larger sample size, and measuring other symptoms associated with ASD are needed to investigate microbial temporal variability in children with ASD as well as the impact of environmental factors and the importance for health outcomes of this variability.

Changes in SCFA concentrations, specifically acetate, propionate, butyrate, isovalerate and valerate, in children with ASD were observed over time, but no changes were observed in CONT children. These variations could be due to changes in substrate availability, in the capacity of the bacteria to produce SCFAs or SCFA absorption by the host. Here, we did not find significant changes in the mmDA and BCoAT genes in children with ASD, suggesting that the decrease in fecal SCFA concentrations could be due to increased absorption by the host, as opposed to decreased production by the bacteria. Previous research has indicated that considering stability based on microbiota function is important (54), as compositional changes might not reflect functional changes due to functional redundancy (55). Changes in microbial metabolites, such as SCFAs, could have important health implications since associations between host health and SCFAs have been established (56) and higher levels of SCFAs were observed in children with ASD (57). Thus, it could be proposed that fluctuations in SCFA concentrations could be associated with host responses due to the impact of SCFAs on GI health (32) and the potential as serving as a messenger in the communication between the GI microbiota and brain (58). Since changes in social deficit or GI symptoms were not detected herein, future studies are needed to elucidate the potential physiological importance of SCFA fluctuations in children with ASD.

Exploring the impact of diet on the microbial stability in children with ASD is important in order to identify potential modifiable environmental influences in promoting microbial stability. In children with ASD who were also below the median in the healthier DP1, a reduction of species diversity was observed, suggesting that the diversity within an individual in this cohort could be more impacted in children scoring low on a healthy eating pattern. Moreover, community structure measured by weighted UniFrac distance tended to change more in children above the median in DP2, indicating the potential that an unhealthier dietary pattern rich in animal-based and processed foods might have a more significant impact on the relative abundance of bacteria in this population. This observation could support previous literature suggesting that the relative abundance of bacterial taxa that are persistently present in the GI tract might be a more significant contributor to temporal dynamics (3). Furthermore, animal-based diets increased β-diversity in humans (15) and individuals following a Western-diet had a decreased diversity compared to individuals following a plant-based diet (59).

Although only a few bacterial taxa were changed in association with dietary patterns in children with ASD, some of the shifts were distinct for each dietary pattern. For example, a decreased intake of healthy foods, such as fruit and vegetables, in children above the median in DP1 coincided with a trend for an increase in potentially pathogenic bacteria such as *Erysipelotrichaceae*. *Erysipelotrichaceae* could be correlated with inflammation (60) and immunomodulation that drives intestinal disease such as colitis (61) and was more abundant in patients with intestinal diseases (e.g., Crohn's Disease, *Clostridium difficile* infection) (62). Furthermore, differences in some SCFA concentrations were observed based on dietary patterns, suggesting that the overall the functional capability to produce SCFA could potentially also be affected by habitual dietary intake in children with ASD.

Overall, less variations than expected were observed in overall structure and abundance of bacterial taxa in this population of children with ASD. The lack of significant changes in individual bacterial taxa could be explained by the relative stability of the microbiota based on α- and β- diversity matrices, as well as the small sample size, the limited amount of samples collected for each participant and the time between collection time points. Additionally, since no associations with GI symptoms or social deficit scores were observed throughout the analyses, the physiological relevance of the temporal variability observed in microbial taxa and metabolites remains to be determined. The lack of association with could potentially be explained by the relative stability of ASD symptoms over time (63) as well as the potential that the microbiota could potentially have a stronger impact on other symptoms of ASD (i.e., repetitive behaviors) as well as on associated symptoms (e.g., irritability). Nevertheless, the preliminary data reported herein can inform future studies including a larger number of subjects with more frequent stool sampling and measuring other symptoms of ASD to further identify the microbial stability in this population and to analyze how microbial variability potentially impacts symptomology.

Despite these limitations, the results reported herein provide preliminary evidence that the microbiota composition of children with ASD could vary over time and that diet might play an important role in determining stability of microbiota in this population. Future studies investigating the longitudinal dynamics in the GI microbiota and factors contributing to stability or instability are warranted. Our analyses are strengthened by including factors known to influence the microbiota composition, such as changes in dietary intake and medication (64) as covariates in the longitudinal analysis. To the best of our knowledge, this is the first study investigating longitudinal dynamics of the GI microbiota in children with ASD. Understanding the environmental factors that determine the functional and compositional changes in the GI microbiota could provide important evidence for the development of intervention strategies for microbiota-associated diseases such as ASD.

## Data Availability Statement

The raw data supporting the conclusions of this manuscript will be made available by the authors, without undue reservation, to any qualified researcher.

## Ethics Statement

This study was carried out in accordance with the recommendations of protocol for human subject research of the Institutional Review Board of the University of Illinois at Urbana-Champaign with written informed consent from all subjects. All subjects gave written informed consent in accordance with the Declaration of Helsinki. The protocol was approved by the name of committee.

## Author Contributions

KB contributed to the design of the study, and was responsible the acquisition, analysis and interpretation of data for the work. She prepared the first draft and approved the final version of the manuscript. SD contributed to the design of the study, interpretation of data for the work and reviewed, and approved the final version of the manuscript.

### Conflict of Interest

The authors declare that the research was conducted in the absence of any commercial or financial relationships that could be construed as a potential conflict of interest.

## References

[1] Kaczmarek JL Musaad SM Holscher HD Time of day and eating behaviors are associated with the composition and function of the human gastrointestinal microbiota. Am J Clin Nutr. (2017) 75:673–82. 10.3945/ajcn.117.156380 28971851

[2] Caporaso JG Lauber CL Costello EK Berg-Lyons D Gonzalez A Stombaugh J. Moving pictures of the human microbiome. Genome Biol. (2011) 12:R50. 10.1186/gb-2011-12-5-r50 21624126 PMC3271711

[3] Flores GE Caporaso JG Henley JB Rideout JR Domogala D Chase J. Temporal variability is a personalized feature of the human microbiome. Genome Bio. (2014) 15:531. 10.1186/s13059-014-0531-y 25517225 PMC4252997

[4] Holling SC Resilience and stability of ecological systems. An Rev Ecol System. (1973) 4:1–23. 10.1146/annurev.es.04.110173.000245

[5] Bäckhed F Fraser CM Ringel Y Sanders ME Sartor RB Sherman PM. Defining a healthy human gut microbiome: current concepts, future directions, and clinical applications. Cell Host Microbe. (2012) 12:611–22. 10.1016/j.chom.2012.10.012 23159051

[6] Coyte KZ Schluter J Foster KR. The ecology of the microbiome: networks, competition, and stability. Science. (2015) 350:663–6. 10.1126/science.aad2602 26542567

[7] Galloway-Peña JR Smith DP Sahasrabhojane P Wadsworth WD Fellman BM Ajami NJ. Characterization of oral and gut microbiome temporal variability in hospitalized cancer patients. Genome Med. (2017) 9:21. 10.1186/s13073-017-0409-1 28245856 PMC5331640

[8] Li K Bihan M Methé BA. Analyses of the stability and core taxonomic memberships of the human microbiome. PLoS ONE. (2013) 8:e63139. 10.1371/journal.pone.0063139 23671663 PMC3646044

[9] Faith JJ Guruge JL Charbonneau M Subramanian S Seedorf H Goodman AL. The long-term stability of the human gut microbiota. Science. (2013) 341:1237439. 10.1126/science.1237439 23828941 PMC3791589

[10] Jalanka J Characterization of intestinal microbiota in healthy adults and the effect of perturbations. Helsinki: University of Helsinki. Doctroal Dissertation (2014).

[11] Ley RE Hamady M Lozupone C Turnbaugh PJ Ramey RR Bircher JS. Evolution of mammals and their gut microbes. Science. (2008) 320:1647–51. 10.1126/science.1155725 18497261 PMC2649005

[12] Mättö J Maunuksela L Kajander K Palva A Korpela R Kassinen A. Composition and temporal stability of gastrointestinal microbiota in irritable bowel syndrome—a longitudinal study in IBS and control subjects. FEMS Immunol Med Microbiol. (2005) 43:213–22. 10.1016/j.femsim.2004.08.009 15747442

[13] Maukonen J Satokari R Mättö J Söderlund H Mattila-Sandholm T Saarela M. Prevalence and temporal stability of selected clostridial groups in irritable bowel syndrome in relation to predominant faecal bacteria. J Med Microbiol. (2006) 55:625–33. 10.1099/jmm.0.46134-0 16585652

[14] Scanlan PD Shanahan F O'Mahony C Marchesi JR. Culture-independent analyses of temporal variation of the dominant fecal microbiota and targeted bacterial subgroups in Crohn's disease. J Clin Microbiol. (2006) 44:3980–8. 10.1128/JCM.00312-06 16988018 PMC1698357

[15] David LA Maurice CF Carmody RN Gootenberg DB Button JE Wolfe BE Diet rapidly and reproducibly alters the human gut microbiota. Nature. (2014) 505:559–63. 10.1038/nature12820 24336217 PMC3957428

[16] Li J Hou Q Zhang J Xu H Sun Z Menghe B. Carbohydrate staple food modulates gut microbiota of Mongolians in China. Front Microbiol. (2017) 8:484. 10.3389/fmicb.2017.00484 28377764 PMC5359301

[17] Wu GD Chen J Hoffmann C Bittinger K Chen YY Keilbaugh SA. Linking long-term dietary patterns with gut microbial enterotypes. Science. (2011) 334:105–8. 10.1126/science.1208344 21885731 PMC3368382

[18] Albenberg LG Wu GD. Diet and the intestinal microbiome: associations, functions, and implications for health and disease. Gastroenterology. (2014) 146:1564–72. 10.1053/j.gastro.2014.01.058 24503132 PMC4216184

[19] Berding K Holscher HD Arthur AE Donovan SM. Fecal microbiome composition and stability in 4-to 8-year old children is associated with dietary patterns and nutrient intake. J Nutr Biochem. (2018) 56:165–74. 10.1016/j.jnutbio.2018.01.002 29571010

[20] Berding K Donovan SM. Diet can impact microbiota composition in children with autism spectrum disorder. Front Neurosci. (2018) 12:515. 10.3389/fnins.2018.00515 30108477 PMC6079226

[21] Tomova A Husarova V Lakatosova S Bakos J Vlkova B Babinska K. Gastrointestinal microbiota in children with autism in Slovakia. Physiol Behav. (2015) 138:179–87. 10.1016/j.physbeh.2014.10.033 25446201

[22] Li M Bauer LL Chen X Wang M Kuhlenschmidt TB Kuhlenschmidt MS. Microbial composition and in vitro fermentation patterns of human milk oligosaccharides differ between formula-fed and sow-reared piglets. J Nutr. (2012) 142:681–9. 10.3945/jn.111.154427 22399522 PMC3301989

[23] Muturi EJ Donthu RK Fields CJ Moise IK Kim CH. Effect of pesticides on microbial communities in container aquatic habitats. Sci Rep. (2017) 7:44565. 10.1038/srep44565 28300212 PMC5353589

[24] Caporaso JG Kuczynski J Stombaugh J Bittinger K Bushman FD Costello EK. QIIME allows analysis of high-throughput community sequencing data. Nat Methods. (2010) 7:335–6. 10.1038/nmeth.f.303 20383131 PMC3156573

[25] Bokulich NA Subramanian S Faith JJ Gevers D Gordon JI Knight R. Quality-filtering vastly improves diversity estimates from Illumina amplicon sequencing. Nat Methods. (2013) 10:57–9. 10.1038/nmeth.2276 23202435 PMC3531572

[26] Cohen IL PDDBI-SV: PDD Behavior Inventory – Screening Version. Lutz, FL: Psychological Assessment Resources, Inc. PAR Inc (2011).

[27] Adams JB Johansen LJ Powell LD Quig D Rubin RA. Gastrointestinal flora and gastrointestinal status in children with autism–comparisons to typical children and correlation with autism severity. BMC Gastroenterol. (2011) 11:22. 10.1186/1471-230X-11-22 21410934 PMC3072352

[28] Lewis SJ Heaton KW. Stool form scale as a useful guide to intestinal transit time. Scand J Gastroenterol. (1997) 32:920–4. 10.3109/00365529709011203 9299672

[29] Bandini LG Anderson SE Curtin C Cermak S Evans EW Scampini R. Food selectivity in children with autism spectrum disorders and typically developing children. J Pediatr. (2010) 157:259–64. 10.1016/j.jpeds.2010.02.013 20362301 PMC2936505

[30] Shade A Caporaso JG Handelsman J Knight R Fierer N. A meta-analysis of changes in bacterial and archaeal communities with time. ISME J. (2013) 7:1493–506. 10.1038/ismej.2013.54 23575374 PMC3721121

[31] Kang DW Park JG Ilhan ZE Wallstrom G LaBaer J Adams JB. Reduced incidence of Prevotella and other fermenters in intestinal microflora of autistic children. PLoS ONE. (2013) 8:e68322. 10.1371/journal.pone.0068322 23844187 PMC3700858

[32] Claesson MJ Jeffery IB Conde S Power SE O'Connor EM Cusack S. Gut microbiota composition correlates with diet and health in the elderly. Nature. (2012) 488:178. 10.1038/nature11319 22797518

[33] Khachatryan ZA Ktsoyan ZA Manukyan GP Kelly D Ghazaryan KA Aminov RI. Predominant role of host genetics in controlling the composition of gut microbiota. PLoS ONE. (2008) 3:e3064. 10.1371/journal.pone.0003064 18725973 PMC2516932

[34] Ledford JR Gast DL Feeding problems in children with autism spectrum disorders a review. Focus Autism Other Dev Disabil. (2006) 21:153–66. 10.1177/10883576060210030401

[35] Marí-Bauset S Llopis-González A Zazpe I Marí-Sanchis A Suárez-Varela MM. Nutritional impact of a gluten-free casein-free diet in children with autism spectrum disorder. J Autism Dev Disord. (2016) 46:673–84. 10.1007/s10803-015-2582-7 26428353

[36] Kong HH Oh J Deming C Conlan S Grice EA Beatson MA. Temporal shifts in the skin microbiome associated with disease flares and treatment in children with atopic dermatitis. Genome Res. (2012) 22:850–9. 10.1101/gr.131029.111 22310478 PMC3337431

[37] Li L Su Q Xie B Duan L Zhao W Hu D. Gut microbes in correlation with mood: case study in a closed experimental human life support system. Neurogastroenterol Motil. (2016) 28:1233–40. 10.1111/nmo.12822 27027909

[38] Huttenhower C Gevers D Knight R Abubucker S Badger JH Chinwalla AT Structure, function and diversity of the healthy human microbiome. Nature. (2012) 486:207 10.1038/nature11234 22699609 PMC3564958

[39] Costello EK Lauber CL Hamady M Fierer N Gordon JI Knight R. Bacterial community variation in human body habitats across space and time. Science. (2009) 326:1694–7. 10.1126/science.1177486 19892944 PMC3602444

[40] Martínez I Muller CE Walter J. Long-term temporal analysis of the human fecal microbiota revealed a stable core of dominant bacterial species. PLoS ONE. (2013) 8:e69621. 10.1371/journal.pone.0069621 23874976 PMC3712949

[41] De Angelis M Piccolo M Vannini L Siragusa S De Giacomo A Serrazzanetti DI Fecal microbiota and metabolome of children with autism and pervasive developmental disorder not otherwise specified. PLoS ONE. (2013) 8:e76993 10.1371/journal.pone.0076993 24130822 PMC3793965

[42] Grimaldi R Cela D Swann JR Vulevic J Gibson GR Tzortzis G. *In vitro* fermentation of B-GOS: impact on faecal bacterial populations and metabolic activity in autistic and non-autistic children. FEMS Microbiol Ecol. (2017) 93:fiw233. 10.1093/femsec/fiw233 27856622 PMC5155555

[43] Finegold SM. Therapy and epidemiology of autism–clostridial spores as key elements. Med Hypotheses. (2008) 70:508–11. 10.1016/j.mehy.2007.07.019 17904761

[44] Williams BL Hornig M Buie T Bauman ML Cho Paik M Wick I. Impaired carbohydrate digestion and transport and mucosal dysbioisis in the intestines of children with autism and gastrotinestinal disturbances. PLoS ONE. (2011) 6:e24585. 10.1371/journal.pone.0024585 21949732 PMC3174969

[45] Darnaud M Dos Santos A Gonzalez P Augui S Lacoste C Desterke C. Enteric delivery of regenerating family member 3 alpha alters the intestinal microbiota and controls inflammation in mice with colitis. Gastroenterology. (2017) 154:1009–23.e14. 10.1053/j.gastro.2017.11.003 29133078

[46] Ma B McComb E Gajer P Yang H Humphrys M Okogbule-Wonodi AC. Microbial biomarkers of intestinal barrier maturation in preterm infants. bioRxiv. (2018) 316257. 10.1101/316257 30487786 PMC6246636

[47] Narushima S Sugiura Y Oshima K Atarashi K Hattori M Suematsu M. Characterization of the 17 strains of regulatory T cell-inducing human-derived Clostridia. Gut Microbes. (2014) 5:333–9. 10.4161/gmic.28572 24642476 PMC4153770

[48] Atarashi K Tanoue T Oshima K Suda W Nagano Y Nishikawa H T reg induction by a rationally selected mixture of Clostridia strains from the human microbiota. Nature. (2013) 500:232 10.1038/nature12331 23842501

[49] Stefka AT Feehley T Tripathi P Qiu J McCoy K Mazmanian SK. Commensal bacteria protect against food allergen sensitization. Proc Natl Acad Sci USA. (2014) 111:13145–50. 10.1073/pnas.1412008111 25157157 PMC4246970

[50] Soomro AH Masud T Anwaar K Role of lactic acid bacteria (LAB) in food preservation and human health-a review. Pakistan J Nutr. (2002) 1:20–4. 10.3923/pjn.2002.20.24

[51] Lukjancenko O Ussery DW Wassenaar TM. Comparative genomics of Bifidobacterium, Lactobacillus and related probiotic genera. Microb Ecol. (2012) 63:651–73. 10.1007/s00248-011-9948-y 22031452 PMC3324989

[52] Nouaille S Rault L Jeanson S Loubière P Le Loir Y Even S. Contribution of Lactococcus lactis reducing properties to the downregulation of a major virulence regulator in *Staphylococcus aureus*, the agr system. Appl Environ Microbiol. (2014) 80:7028–35. 10.1128/AEM.02287-14 25192992 PMC4248995

[53] Bajer L Kverka M Kostovcik M Macinga P Dvorak J Stehlikova Z. Distinct gut microbiota profiles in patients with primary sclerosing cholangitis and ulcerative colitis. World J Gastroenterol. (2017) 23:4548. 10.3748/wjg.v23.i25.4548 28740343 PMC5504370

[54] Lozupone CA Stombaugh JI Gordon JI Jansson JK Knight R. Diversity, stability and resilience of the human gut microbiota. Nature. (2012) 489:220–30. 10.1038/nature11550 22972295 PMC3577372

[55] Moya A Ferrer M. Functional redundancy-induced stability of gut microbiota subjected to disturbance. Trends Microbiol. (2016) 24:402–13. 10.1016/j.tim.2016.02.002 26996765

[56] Macfarlane GT Macfarlane S. Bacteria, colonic fermentation, and gastrointestinal health. AOAC Int. (2012) 95:50–60. 10.5740/jaoacint.SGE_Macfarlane 22468341

[57] Wang L Christophersen CT Sorich MJ Gerber JP Angley MT Conlon MA. Elevated fecal short chain fatty acid and ammonia concentrations in children with autism spectrum disorder. Dig Dis Sci. (2012) 57:2096–102. 10.1007/s10620-012-2167-7 22535281

[58] Berding K Donovan SM. Microbiome and nutrition in autism spectrum disorder: current knowledge and research needs. Nutr Rev. (2016) 74:723–36. 10.1093/nutrit/nuw048 27864534

[59] Conlon MA Bird AR. The impact of diet and lifestyle on gut microbiota and human health. Nutrients. (2014) 7:17–44. 10.3390/nu7010017 25545101 PMC4303825

[60] Kaakoush NO. Insights into the role of erysipelotrichaceae in the human host. Front Cell Infect Microbiol. (2015) 5:84. 10.3389/fcimb.2015.00084 26636046 PMC4653637

[61] Palm NW de Zoete MR Cullen TW Barry NA Stefanowski J Hao L. Immunoglobulin A coating identifies colitogenic bacteria in inflammatory bowel disease. Cell. (2014) 158:1000–10. 10.1016/j.cell.2014.08.006 25171403 PMC4174347

[62] Mancabelli L Milani C Lugli GA Turroni F Cocconi D van Sinderen D. Identification of universal gut microbial biomarkers of common human intestinal diseases by meta-analysis. FEMS Microbiol Ecol. (2017) 93:fix153. 10.1093/femsec/fix153 29126267

[63] Bieleninik Ł Posserud MB Geretsegger M Thompson G Elefant C Gold C. Tracing the temporal stability of autism spectrum diagnosis and severity as measured by the Autism Diagnostic Observation Schedule: a systematic review and meta-analysis. PLoS ONE. (2017) 12:e0183160. 10.1371/journal.pone.0183160 28934215 PMC5608197

[64] Maier L Typas A. Systematically investigating the impact of medication on the gut microbiome. Curr Opin Microbiol. (2017) 39:128–35. 10.1016/j.mib.2017.11.001 29169088

