# Supplementary material for: Dietary Patterns Impact Temporal Dynamics of Fecal Microbiota Composition in Children With Autism Spectrum Disorder
Source: Front Nutr. 2020 Jan 10;6:193. doi: 10.3389/fnut.2019.00193 (PMC6968728; doi:10.3389/fnut.2019.00193)
Supplement: Supplementary file 2 [file Table_2.docx]

**Supplemental Table 2**. Bacterial abundance and food groups contributing to stability categories in CONT

|  | **Less stable (n=13)** | **More stable (n=13)** |
| --- | --- | --- |
| **Bacterial richness/diversity** |  |  |
| Shannon Index | 4.9 (4.3-5.5) | 5.2 (4.8-5.6)† |
| **Bacterial abundance (% of sequences**) |  |  |
| Verrucomicrobia | 1.88 (0.03-10.8) | 0.4 (0.01-1.3)* |
| *Adlercreutzia* | 0.004 (0-0.07) | 0.06 (0.001-0.19)* |
| *Faecalibacterium* | 8.7 (5.9-12.8) | 12.9 (8.5-18.4)* |
| *Sutterella* | 0.26 (0.003-0.63) | 0.31 (0.13-1.65)† |
| *Bilophila* | 0 (0-0.1) | 0.1 (0-0.07)† |
| *Akkermansia* | 1.88 (0.03-10.7) | 0.39 (0.01-1.3)* |
| **Food group (servings/day)^1^** |  |  |
| Vegetables | 2.0 (1.8-3.2) | 2.5 (1.8-3.1)† |
| Sweetened beverages | 0.12 (0.08-1.7) | 0.18 (0.08-0.26)† |
| Kid’s meals | 1.6 (1.1-2,1) | 0.9 (0.5-1.3)* |
| Fish | 0.06 (0.06-0.3) | 0.18 (0.12-0.3)* |
| Condiments | 0.16 (0.08-0.3) | 0 (0-0.3)* |
| **Nutrient^2^** |  |  |
| Whole grains (g) | 0.84 (0.35-1.45) | 0.95 (0.39-2.5)† |

Data expressed as median (IQR); FDR-corrected *p≤0.05; †p≤0.1; Individuals in the CONT group were assigned to a category of stability based on falling above or below the median of median weighted UniFrac distances

^1^Food groups derived from food frequency questionnaire

^2^Nutrient intake derived from 3-day food diary
