# Supplementary material for: Dietary Patterns Impact Temporal Dynamics of Fecal Microbiota Composition in Children With Autism Spectrum Disorder
Source: Front Nutr. 2020 Jan 10;6:193. doi: 10.3389/fnut.2019.00193 (PMC6968728; doi:10.3389/fnut.2019.00193)
Supplement: Supplementary file 3 [file Table_3.DOCX]

**Supplemental Table 3.** Variation of coefficient of variation (CV) based on dietary patterns in children with ASD

|  | **Dietary Pattern 1** | | **Dietary Pattern 2** | |
| --- | --- | --- | --- | --- |
| **Measure** | **Above median (n=13)** | **Below median (n=13)** | **Above median (n=13)** | **Below median (n=13)** |
| Chao1 Index CV | 0.05 (00.04-0.08) | 0.05 (0.03-0.13) | 0.05 (0.02-0.09) | 0.05 (0.04-0.11) |
| Observed OTUs CV | 0.08 (0.06-0.14) | 0.08 (0.02-0.18) | 0.07 (0.03-0.12) | 0.08 (0.05-0.14) |
| Shannon Index CV | 0.06 (0.05-0.09) | 0.08 (0.04-0.14) | 0.07 (0.06-0.1) | 0.07 (0.03-0.13) |
| Simpson Index CV | 0.03 (0.01-0.04) | 0.03 (0.01-0.04) | 0.02 (0.01-0.03) | 0.03 0.01-0.04) |

Data expressed as Median (IQR); Dietary patterns were derived from the Youth and Adolescence Food Frequency Questionnaire using principal component and factor analysis.
