# Supplementary figures and images for: Dietary Patterns Impact Temporal Dynamics of Fecal Microbiota Composition in Children With Autism Spectrum Disorder
Source: Front Nutr. 2020 Jan 10;6:193. doi: 10.3389/fnut.2019.00193 (PMC6968728; doi:10.3389/fnut.2019.00193)

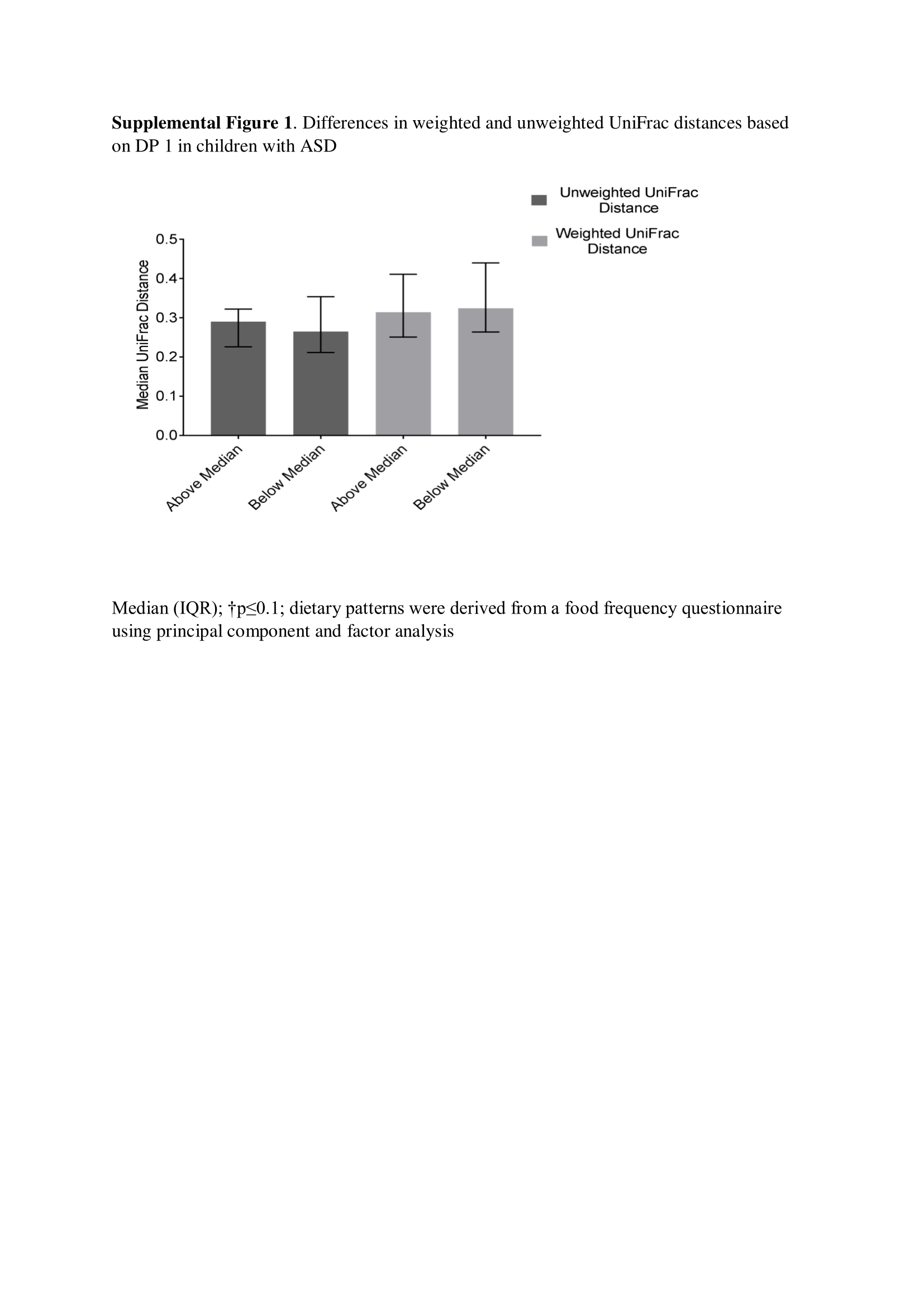

Supplement: Supplementary file 4 [file Image_1.TIFF]
